# Cell surface engineering and application in cell delivery to heart diseases

**DOI:** 10.1186/s13036-018-0123-6

**Published:** 2018-12-04

**Authors:** Daniel Y. Lee, Byung-Hyun Cha, Minjin Jung, Angela S. Kim, David A. Bull, Young-Wook Won

**Affiliations:** Division of Cardio-Thoracic Surgery, Department of Surgery, University of Arizona College of Medicine, Room 4302D, 1501 N Campbell Ave, Tucson, Arizona 85724 USA

**Keywords:** Cell surface engineering, cell modification, mesenchymal stem cells, cell therapy, cardiac diseases, cardiac repair

## Abstract

Cell-based therapy has expanded its influence in cancer immunotherapy, regenerative medicine, and tissue engineering. Due to their secretory functions, differentiation capabilities, specific homing effects through chemotaxis, distinctive therapeutic potentials, and *ex vivo* expandability, cells have become an attractive reagent for advanced therapeutic strategies. Therefore, the ability to modify cells and manipulate their functions according to intended therapeutic designs has been the central scientific interest in the field of biomedical research. Many innovative methods have been developed with genetic modification of cells being the most advanced cell surface engineering technique. Although genetic modification is a powerful tool, it has a limited applicability due to the permanent modifications made on cells. Alternatively, many endeavors have been made to develop surface engineering techniques that can circumvent the limitations of genetic modification. In this review, current methods of non-genetic cell surface modification, including chemical conjugations, polymeric encapsulation, hydrophobic insertion, enzymatic and metabolic addition, will be introduced. Moreover, cell surface engineering plausible for cardiac remodeling and the future prospective will be discussed at the end.

## Background

Cell surface engineering to provide new characteristics and functions to cells has drawn continuous interest from researchers in biomedical science as cell therapy has emerged as a prominent therapeutic strategy equivalent and complementary to the conventional therapeutic approaches. Research endeavors over the past several decades have identified various types of cells as suitable living drugs and versatile drug carriers. In particular, stem cells, including mesenchymal stem cells (MSCs), hematopoietic stem cells (HSCs), and induced pluripotent stem cells (iPSCs), and immune cells, such as T-cells and Natural Killer (NK) cells, have been favored candidates for regenerative medicine and cell-based cancer immunotherapy, respectively. Both bone marrow-derived and adipose-derived MSCs readily isolated from the body are able to release cytokines and growth factors that can be utilized towards wound healing, treating cardiovascular diseases, and correcting neurological disorders [1–7]. Adoptive transfer of *ex vivo* cultured and activated immune cells isolated from cancer patients has shown refreshing clinical results [8, 9]. Unfortunately, these breakthrough discoveries in both regenerative medicine and cancer immunotherapy using cells as therapeutic reagents soon faced a common problem: the inability to control cellular functions to maximize the therapeutic benefits. MSCs directly injected into the myocardium showed low retention rate with only 0.44% of the transplanted MSCs remaining in the myocardium after 4 days of administration [10]. Moreover, systemic injection of MSCs on rat myocardial infarction (MI) models revealed less than 1% accumulation of MSCs in the ischemic myocardium [11]. To overcome the low retention rates and enhance the target homing effect, MSCs were genetically engineered to overexpress CXC chemokine receptor 4 (CXCR4), a receptor for stromal-derived factor-1 (SDF-1) expressed in injured myocardium [12]. The resulting genetically modified MSCs showed enhanced target homing effect and greater retention rate in the ischemic myocardium after the intravenous delivery. The developmental story of cell-based cancer immunotherapy is not so different from MSCs in regenerative medicine. Although the efficacy of adoptive transfer of tumor infiltrating lymphocytes (TILs) was examined over several decades, genetically engineered T cells expressing chimeric antigen receptors (CARs) rapidly replaced the application of TILs due to their high specificity, non-MHC-restricted recognition of tumor antigen, superior potency, and improved *in vivo* persistency [9, 13, 14].

Early attempts to control the cellular interactions and reprogramming the cellular functions focused on the *ex vivo* preconditioning [15, 16]. In this method, multiple stimuli, including pharmacological agents, cytokines, stimulatory ligands, and/or microenvironmental preconditioning, are challenged to the cells of interest in order to achieve enhanced cell survival, differentiation, paracrine effects, specificity, potency, and target homing effect. For instance, hypoxic conditioning increased the expression of pro-survival and pro-angiogenic factors on MSCs and improved their potential to repair the injured myocardium [17, 18]. Many *ex vivo* immune cell expansion and activation protocols also require addition of cytokines, such as interleukin (IL)-2, IL-12, IL-15, IL-18, and IL-2, to the culture media [15, 19]. Although preconditioning methods improved the *in vivo* cell retention and survival, they only allowed minimal gain of control to manipulate the cellular functions that is necessary to redirect cells for therapeutic purposes. As cell therapy continues to evolve, preconditioning methods have been integrated as essential protocols for the growth and maintenance of cells cultured in *ex vivo* conditions, and many creative methods have been developed to improve the therapeutic feasibility and effectiveness of cells.

Genetic engineering, currently the state-of-the-art modification techniques, has opened up new avenues to tailor preexisting cells to acquire specific therapeutic functions. The most celebrated example is the aforementioned CAR-T cells. Recently, the United States Food and Drug Administration (FDA) approved two CAR-T cells, Kymriah™ and Yescarta™, for the treatment of B cell precursor acute lymphoblastic leukemia (BCP-ALL) and large B cell lymphoma [20]. Both CAR-T cells are engineered to express CARs specific for CD19 expressed on normal and malignant B lineage cells. Genetic engineering also extends its application to modify MSCs by overexpressing receptors and proteins for regenerative medicine: CXCR4 to take advantage of SDF-1 chemotaxis; fibroblast growth factor-2 (FGF2) for improved viability after transplantation into injured myocardium; heme oxygenase-1 (HO-1) to improve cell survival, organ recovery, and function in injured heart; and vascular endothelial growth factor (VEGF) for angiogenesis and inhibition of progression of left ventricular hypertrophy [21, 22]. Undoubtedly, genetic engineering is a powerful tool to control the cellular function of cells; however, it has several drawbacks requiring profound consideration for incorporation into the therapeutic designs. The major drawback is the use of viral vectors to deliver therapeutic genes into the cells of interest [21, 23–26]. Viral vectors have higher risk of genetic integration that may lead to tumorigenesis and trigger immunogenic response [27]. Additional features introduced to cells through viral genetic engineering are permanent and irreversible, exacerbating the safety risk in clinical settings [28, 29]. Non-viral gene carriers alleviate the safety concerns; however, they show rather low transfection efficiency compared to viral vectors [30]. Because the success of genetic engineering heavily depends on the transduction/transfection efficiency, the resulting modified cells may show inconsistent and unpredictable therapeutic efficacy. This is because genetic engineering is not applicable to all types of cells, especially stem cells and slowly dividing cells.

Alternative to genetic engineering, non-genetic cell surface engineering techniques, such as covalent conjugation [31–34], electrostatic interactions [35–37], hydrophobic insertion [38–43], offer more transient and reversible modifications to control cellular functions. Instead of manipulating cells at the gene and protein level, these techniques modify the cell using the characteristics of lipids, proteins, and glycans present in the cell membrane [5, 29, 44, 45]. Because those are essential components for cells, non-genetic surface engineering techniques can potentially be applied to a broad range of cells from different origins. Through non-genetic cell surface engineering, biomaterials including proteins, surface receptors, antibodies, peptides, genetic materials, and protective polymers, have been used to endow specific functions to cells [31, 33, 34, 42, 44, 46–48]. Research areas that have benefitted from these cell surface modifications include (1) investigation of adding new functions, (2) reducing graft rejection for transplantation by masking the surface antigens, (3) creation of heterogeneous cluster of cells by cell-to-cell attachment, (4) enhancing immune effector functions, and (5) programing cell-to-cell interactions.

Ideal cell surface engineering methods should provide control over the fate and function of the modified cells without interfering with cell survival, proliferation, and cellular activities. Therefore, this review attempts to provide a concise guide on cell surface engineering techniques that meet the purpose of modifying the cell surface properties. The first section summarizes each type of non-genetic cell surface engineering technique with application on different cell types. In the following section, challenges and considerations of engineering the surface of living cells are discussed. Finally, example of cell surface engineering technique is presented as a promising method to redirect MSCs for cardiac diseases.

## Non-genetic Surface Engineering

### Covalent Conjugation

**Fig. 1:**
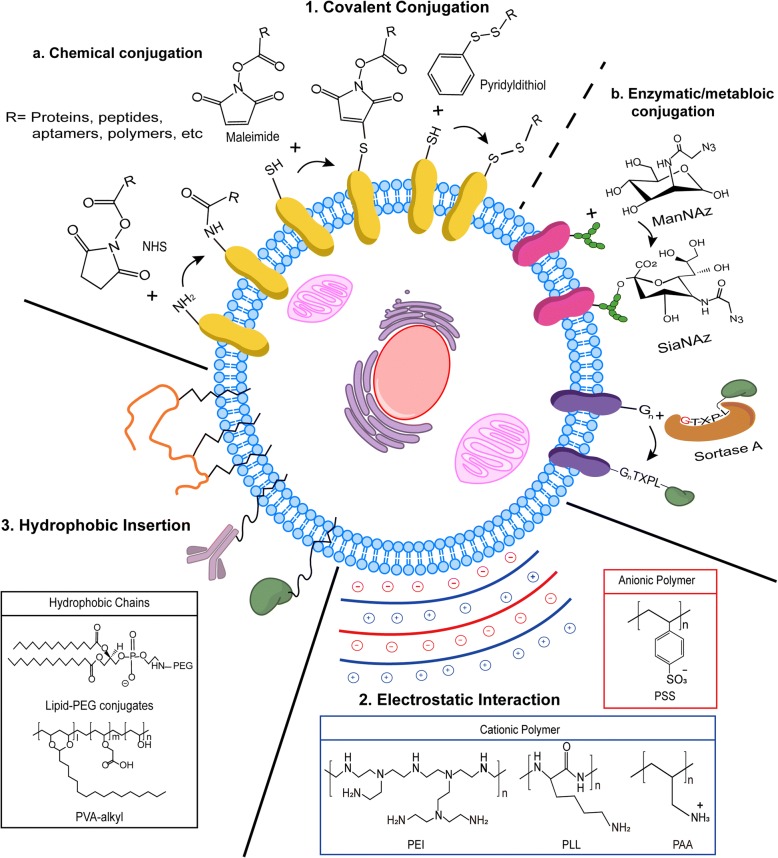
Modes of non-genetic cell surface engineering techniques. (1) Incorporation of cross-linkers, such as NHS, Maleimide, or pyridyldithiol, allows cell surface modification with biomaterials through chemical covalent conjugation. Cell metabolism of unnatural sugar and enzymatic reactions can be exploited to attach functional groups on the cell surface. (2) Electrostatic interactions between the cell surface and the charged polymers such as PEI, PLL, PAA, and PSS can modify cells through layer-by-layer technique. Also, charged block-co-polymers, such as PLL-PEG, can modify the cell surface through electrostatic interaction. (3) Lipid-conjugated bioactive molecules or polymers with long alkyl chains can be embedded into the cell membrane through hydrophobic interaction. Abbreviations: NHS: *N*-hydroxyl-succinimidyl ester; ManNAz: *N*-α-azidoacetylmannosamine; PAA: Poly(acrylic acid); PEG: Poly(ethylene glycol); PEI: Poly(ethyleneimine); PLL: Poly-L-lysine; PSS: Poly(styrene) sulfate; PVA: Poly(vinyl alcohol); SiaNAz: *N*-α-azidoacetyl sialic acid

Covalent conjugation chemically, metabolically, or enzymatically attaches bioactive substances to the cell membrane [31, 33, 34, 48–51]. Chemical conjugation is the most straightforward method that takes advantage of surface-exposed functional groups on the membrane proteins as grafting points. Currently, *N-*hydroxyl-succinimidyl ester (NHS) groups [31, 33, 34, 48], maleimide [51], and pyridyldithiol [52, 53] are the most frequently used chemical cross-linkers (Fig. 1). The use of NHS-activated esters modifies exposed amine groups on the surface of bioactive molecules. Maleimide conjugated biomolecules can be selectively attached to surface-exposed thiol group, generating a non-cleavable thio-ether bond. In case degradable conjugation is desired, pyridyldithiol modified biomolecules can be attached to free thiols on the surface to create reducible disulfide bonds. The key advantage of chemical conjugation is the broad applicability. Biomaterials functionalized with cross-linkers can be used to modify variety of cells. Unlike the random modification through chemical conjugation, metabolic and enzymatic conjugation methods provide more selective attachment of biomaterials. Saxon et al. and Prescher et al. reported the use of sophisticated metabolic surface modification that takes advantage of unnatural sialic acid biosynthesis [49, 50]. Human cells undergo unnatural sialic acid biosynthesis when exposed to unnatural sugar *N*-α-azidoacetylmannosamine (ManNAz), an analog of the native sugar *N*-acetylmannosamine (Fig. 1). This process incorporates *N*-α-azidoacetyl sialic acid (SiaNAz), a metabolite of ManNAz, to the membrane glycoconjugates. The added azide groups further provide attachment points for biomaterials through Staudinger ligation [49, 50] or click-chemistry [54, 55]. Similar to metabolic conjugation, enzymatic conjugation also provides covalent attachment of biomaterials on a designated spot on the cell surface. As reported by Swee et al., transpeptidase sortase A from *Staphylococcus aureus* efficiently conjugates peptides or proteins with LPETG-motif to the N-terminal glycine exposed on the surface of different types of cells (Fig. 1) [56]. Although conjugated biomaterials gradually disappear over time, modifications installed through covalent conjugation are stable compare to other non-genetic surface engineering methods [33, 34, 40]. Moreover, the degree of modification is difficult to control with covalent conjugation, and higher degree of modification using bioactive molecules, both small or large, may cause significant physiological alterations, such as reduction of membrane mobility and diffusion kinetics to the modified cells [38, 44, 57].

### Electrostatic Interaction

Electrostatic interactions modify the cell surface by establishing self-assembled structures between the negatively charged cell surface and cationic polymers (Fig. 1). Cells initially modified with cationic polymers can be engineered again *via* a layer-by-layer technique by sequentially applying anionic and cationic polymers [35, 37, 58–60]. Because modified cells encapsulated by multiple polymeric layers can reduce molecular recognition, the electrostatic layer-by-layer approach has been often investigated in the cell transplantation research [37, 59]. Many cationic/anionic polymers and poly electrolytes, such as poly-L-lysine (PLL), poly(styrene) sulfate (PSS), poly(allylamine hydrochloride) (PAH), poly(diallydimethylammonium) chloride (PDADMAC or PDDA), poly(ethyleneimine) (PEI), polyphosphoric acid (PPP), and poly(acrylic acid) (PAA), and hyaluronic acid (HA) have been used to generate multiple layers on the cell membrane [35, 37, 58–61]. Thickness of the polymer layer can be controlled by changing the number of layers and the new surface properties of the modified cells rely on the polymer characteristics of the outermost layer. However, it should be noted that high charge density of cationic polymers significantly reduces the viability of modified cells [58, 62, 63]. To improve the cell viability after the surface modification, PLL-*graft*-poly(ethylene glycol) (PLL-*g*-PEG) can be introduced to coat the surface cells [59, 64]. Surface modification of PLL-*g*-PEG was further developed to incorporate functional groups, such as biotin, hydrazide, and azide, to capture streptavidin, aldehyde, and cyclooctyne [64]. The main advantage of surface engineering through electrostatic interaction is that cells are protected from the sheer stress and immune response by the non-invasive encapsulation. Biocompatibility of cationic polymers, however, should be resolved in order to be used in cell therapy.

### Hydrophobic insertion

Amphiphilic polymers polymerized with long alkyl chains, such as phospholipid-conjugated PEGs and poly(vinyl alcohol) (PVA), provide noninvasive modifications of the cell surface through hydrophobic interaction (Fig. 1). Similarly, a large number of different cell types have been modified *via* hydrophobic interaction with lipid-conjugated biomaterials for specific function [38–43, 57, 65–70]. Most lipophilic membrane dyes currently available in the market, such as Dil, DiD, DiR, and DiO, are developed upon cell surface modification through hydrophobic interaction. Interaction of lipid-conjugated PEGs with lipid bilayers was examined by Yamamoto et al. using surface plasmon resonance (SPR) spectroscopy [71]. Lipids with different lengths of alkyl chains—1,2-dimyristoyl-sn-glycerol-3-phosphatidylethanolamine (DMPE, 14 carbons), 1,2-dipalmitoyl-sn-glycerol-3-phosphatidylethanolamine (DPPE, 16 carbons), and 1,2-distearoyl-sn-glycerol-3-phosphatidylethanolamine (DSPE, 18 carbons)—were conjugated with PEG (5 kDa) and applied onto the lipid bilayer. Out of all lipid-PEG conjugates, DMPE showed the most rapid incorporation to the membrane. Insertion of DPPE showed concentration-dependent behavior; however, incorporation of DSPE was only observed at high concentration. Dissociation of DMPE was more rapid compared to DPPE when modified lipid bilayer was washed with PBS. No dissociation was observed once DSPE was incorporated into the membrane. Thus, it was noted that longer hydrophobic chains reduce the incorporation rate and the dissociation rate of lipid molecules [71]. Interestingly, fluorescence of FITC-labeled lipid-PEGs was recovered in a few minutes in fluorescent recovery after photobleaching (FRAP) assay [71]. This observation indicates that lipid-PEGs embedded in the lipid bilayer were able to diffuse laterally within the lipid bilayer. Unlike covalent conjugation and electrostatic interaction, surface modification with hydrophobic insertion allows membrane-anchored bioactive molecules to participate in the dynamic movement of cell membrane. Most importantly, cells modified with lipid-conjugated biomaterials showed negligible toxicity, and the modified cells resumed normal cellular activities [65–67]. Instead of preparing the lipid-conjugatedmolecules, modification of cell surface can be achieved by liposomal fusion strategy [72]. Because liposomes are vesicles composed of lipids and lipid-conjugated molecules, large sections of the liposomes containing specialized lipids can be incorporated into the membrane without causing severe toxicity [72–75]. Fate of the lipid-conjugated bioactive molecules has not been fully understood, and the exclusion pathway requires further investigation; however, the endocytosis of membrane-anchored lipid-conjugated biomaterials has not been observed [76]. Lipid-conjugated biomolecules are believed to be released from the cells to the surroundings due to equilibrium differences [40]. Although molecules of interest must be hydrophobized by lipid or alkyl chain conjugation and the retention time on the surface is variable, hydrophobic insertion is an attractive surface engineering technology that offers rapid and nontoxic surface modification to virtually any type of cell.

## Challenges and considerations of engineering the surface of living cells

### Cell membrane dynamics

Cell membrane is in a dynamic state. It is subjected to undergo constant remodeling where most of its components—lipids and membrane proteins—are internalized, degraded, recycled, and replaced [77, 78]. The rate of these processes is highly dependent on the type of lipids and proteins and varies widely from hours to weeks [79]. Cell membrane lipids and proteins are routinely internalized through endocytosis, pinocytosis, and phagocytosis. Due to their size, type, and property, biomaterials that are chemically conjugated, electrostatically adsorbed, or hydrophobically embedded on the membrane, may internalize mostly through endocytosis [80]. The process of endocytosis is initiated as complementary ligands bind to surface receptors or as bioactive substances are absorbed on the cell membrane [81–83]. These events trigger invaginations of small areas containing the receptors and affected regions of cell membrane. Subsequently, the invaginated pockets are closed, and newly formed vesicles are transported to the intracellular compartments. During endocytosis, any molecules and materials on the invaginated cell membrane and in the proximal media will be taken up by the cells, resulting in the loss of desired functions installed *via* surface engineering. Therefore, surface engineering methods should consider cell membrane dynamics in order to improve the surface residence time of the desired biomaterials for prolonged therapeutic effects.

### *In vivo* system

Unlike the *in vitro* experimental settings, *in vivo* environment is an integrated system of many complex mechanical and biochemical interactions. Transplanted or adoptively transferred surface-engineered therapeutic cells are exposed to sheer stress and hemodynamic forces that can strip off the installed surface modification [84]. Migration in the circulation and endothelial transmigration in the tissues, as demonstrated by leukocytes and stem cells, require extensive reshaping of the cell membrane [85, 86]. In the spleen, circulating cells are forced to enter the compact network of sinusoidal capillaries to eliminate damaged and aged cells [87]. In order to compensate for the mechanical stress from the *in vivo* environment, surface-engineered cells must display unaltered membrane flexibility and elasticity. Surface-engineered cells in blood circulation are also exposed to coagulation factors, the complement immune system, and inflammation mediators that drastically reduce duration of therapeutic effects [88, 89]. Macrophages and monocytes of innate immune defense system are often stimulated in response to the bioactive substances on surface-modified cells and subsequently eliminate them from the body by phagocytosis [90]. Immunogenic biomaterials, such as proteins synthesized from bacterial host and antibodies isolated from animals, are opsonized by neutralizing antibodies and are cleared by the innate immune system and complement activation [91–94]. Thus, cell surface modification, regardless of the methods employed, must not sacrifice the membrane flexibility and elasticity but rather provide new functionality in addition to the protection against mechanical and biological challenges for clinical applications.

### Clinical translation

For clinical translation, surface-engineered cells must satisfy several fundamental principles of biocompatibility. Because cells are the most critical component of cell therapy, any modifications applied to the cell surface should not have detrimental effects on cell viability. At any stage of preparation, cell viability should be maintained by changes in pH, osmolality, temperature, pressure, degree of agitation, and exposure to organic solvent [84]. Surface modification should not become a physical barrier that blocks diffusion of necessary nutrients. This is particularity important for islet cell transplantation, where surface-modified islet cells secrete insulin in response to glucose levels [39, 66, 95]. Unless the purpose for surface engineering is to mask the surface antigens during transplantation or adoptive transfer of immune cells—for the sake of reducing the occurrence of graft-versus-host disease (GVHD)—surface proteins and receptors should be exposed on the surface without hindrance to bind growth factors and ligands to signal cell survival, proliferation, and activation. Moreover, surface engineered biomolecules should not reduce the membrane flexibility and elasticity, which are the essential properties of cell membrane that allows cell adhesion, migration, and signaling [96–99]. Lastly, the cost of surface engineering cells for therapeutic purposes must be affordable. Genetic engineered cells, such as CAR-T cells, can be finely tuned to provide personalized cell therapy for many cancers and diseases; however, the cost of treatment is extremely expensive, estimated at $25,000 per treatment [100]. Genetically engineered stem cells are also anticipated to be one of the most expensive treatment options. The high cost arises from the labor-intensive and time-consuming certified process to prepare genetically engineered cells. The surface modification methods discussed earlier have the potential to be applied as an alternative technology to genetic engineering and are more economical with rapid preparation of therapeutic cells.

## Application of hydrophobic insertion for cardiac diseases

### Stem cell delivery for cardiac injury

Cardiac injuries and diseases remain the most common cause of death globally with a higher annual death rate compared to any other causes [101]. The major reason of the highest mortality is that cardiac injury and diseases can progress rapidly, as seen in the cases of acute myocardial infarction (AMI) and MI. Conversely, these cardiac diseases often show lagging progress of cardiac remodeling that frustrates the recovery. Consequently, cardiac hypertrophy and myocardial fibrosis eventually prevail [102–106]. Heart failure and even death may result as a series of catastrophic processes, including cellular injury, mechanical dysfunction, and disruption of structural integrity, occur. Therefore, clinicians and scientists are burdened to develop therapeutic methods to repair and replace the injured cardiomyocytes or associated cells in the infarcted myocardium.

In terms of therapy designed for cardiac injuries and diseases, the treatment options to heal the infarcted cardiac tissue are extremely limited. Currently available therapies for AMI and MI, such as the treatments concentrated on reducing myocardial oxygen needs, extend survival by protecting the remaining cardiomyocytes without addressing the fundamental problem—the loss of cardiomyocytes [107, 108]. Several strategies of cardiac regeneration have emerged from decades of intensive research efforts. Although most of these strategies are still in the early stage, some are beginning to be clinically tested for practicality [109].

Current research in the field of cardiac diseases attempts to stimulate the endogenous regenerative mechanisms *via* cell-based therapies. Many have believed that regenerative therapies employing stem cells, especially MSCs, have enormous potential for clinical applications to treat cardiac diseases [110]. MSCs, being multipotent stem cells, can differentiate into several cell types, such as mesodermal lineage cells and myogenic lineage [111]. These MSC-based therapies for cardiac diseases are achieved by the intermingling of two major components: a cardiomyocyte source as a target for cardiac regeneration; and a non-myocardial tissue acting as a source for regeneration in an effective cardiac environment [112]. Besides the two major components, other influential factors, such as the type of stem cell being used, its proliferative and differentiation capacity, the targeting to localize the damaged site, the route and site of stem cell transplantation, survival capability of the engrafted cells and so forth, should be carefully tweaked to achieve a successful MSC-based therapy [112].

Regenerative medicine for heart diseases using stem cells has been controversial and readers’ discretion is strongly advised [113–115]. One of the most challenged idea is the existence of resident endogenous stem cells or cardiac progenitor cells (CPCs). The current views concur on the fact that c-Kit^+^ CPCs, once thought to show regenerative functions and ability to replace the lost cardiomyocytes due to the cardiac injury through differentiating into cardiomyocytes, are rare and have minimal cardiomyogenic potential [116]. However, the prevailing view on the rarity of resident endogenous stem cells should not discourage the idea of stem cell therapy towards cardiac injury and diseases since several preclinical have shown improved cardiac function after the transplantation of MSCs into infarcted heart and clinical studies have reported modest benefits for patients with ischemic cardiomyopathy [117–120] Evidence suggests that these beneficial recovery and protective effects are indirect contributions of MSCs through paracrine signaling [114, 121, 122]. The transplanted MSCs secrete growth factors, microRNA (miRNAs), immunomodulatory signals, and exosomes in order to promote prosurvival mechanism and encourage restorative effects in the injured myocardium [112, 114]. Although clinical results and mechanism of actions have not been clearly shown, it is difficult to preclude the therapeutic benefits of stem cell delivery for heart diseases due to lack of understanding. To reiterate, the ability of any treatment strategies to compensate for the loss of the functioning cardiomyocytes, even though it may not indicate the physical replacement of cardiomyocytes, is the essence of stem cell therapy for cardiac injury. One way to improve the clinical outcome of stem cell therapy is to develop a competent delivery method that can specifically target the disease site within the therapeutic time window. In this aspect, cell surface engineering offers the means to enhance the targeting effect of MSCs, or any discovered therapeutic cells or stem cells, without altering their native functions.

### SDF-1/CXCR4 on MSCs

At the ischemic sites, MSCs can secrete arteriogenic cytokines, such as vascular endothelial growth factor (VEGF), basic fibroblast growth factor (bFGF), placental growth factor (PIGF), and monocyte chemoattractant protein-1 (MCP-1), to repair the damaged tissues [123, 124]. Thus, many have strived to design a method that allows intravenously infused MSCs to target the injured myocardium. Unfortunately, a negligible number of MSCs migrated to the ischemic myocardium when a large amount of MSCs were intravenously infused [11]. Poor migration of MSCs is related to the loss of CXCR4 expression [125]. *Ex vivo* expansion of MSCs is necessary to generate a therapeutically relevant number of cells; however, MSCs express heterogeneous CXCR4 with significantly reduced affinity to their corresponding ligands, SDF-1, during the expansion. Moreover, Rombouts et al. have reported that *ex vivo* expansion of MSCs results in the loss of CXCR4 expression on MSCs [126]. This effect ultimately reduces the chemotaxis of MSCs along the chemokine gradient to specific sites. Systematic administration of MSCs should therefore be improved with a reliable targeting method to enhance therapeutic efficacy.

Immediately after myocardial infarction, injured cardiomyocytes up-regulate SDF-1 expression to recruit stem cells for repair [127, 128]. Although many studies have stated that migration of CXCR4^+^ bone marrow stem cells along the SDF-1 concentration gradient is critical for cardiac recovery [127, 129, 130], it has been suggested that the responsiveness to SDF-1 in these cells may mature over 4-7 days after MI [131, 132]. Conversely, expression of SDF-1 in the heart starts to decline 4-7 days after the ischemic injury [127]. Thus, expanding autologous MSCs—which takes several weeks—for the treatment of MI is not ideal due to the shallow therapeutic window of SDF-1 expression.

**Fig. 2:**
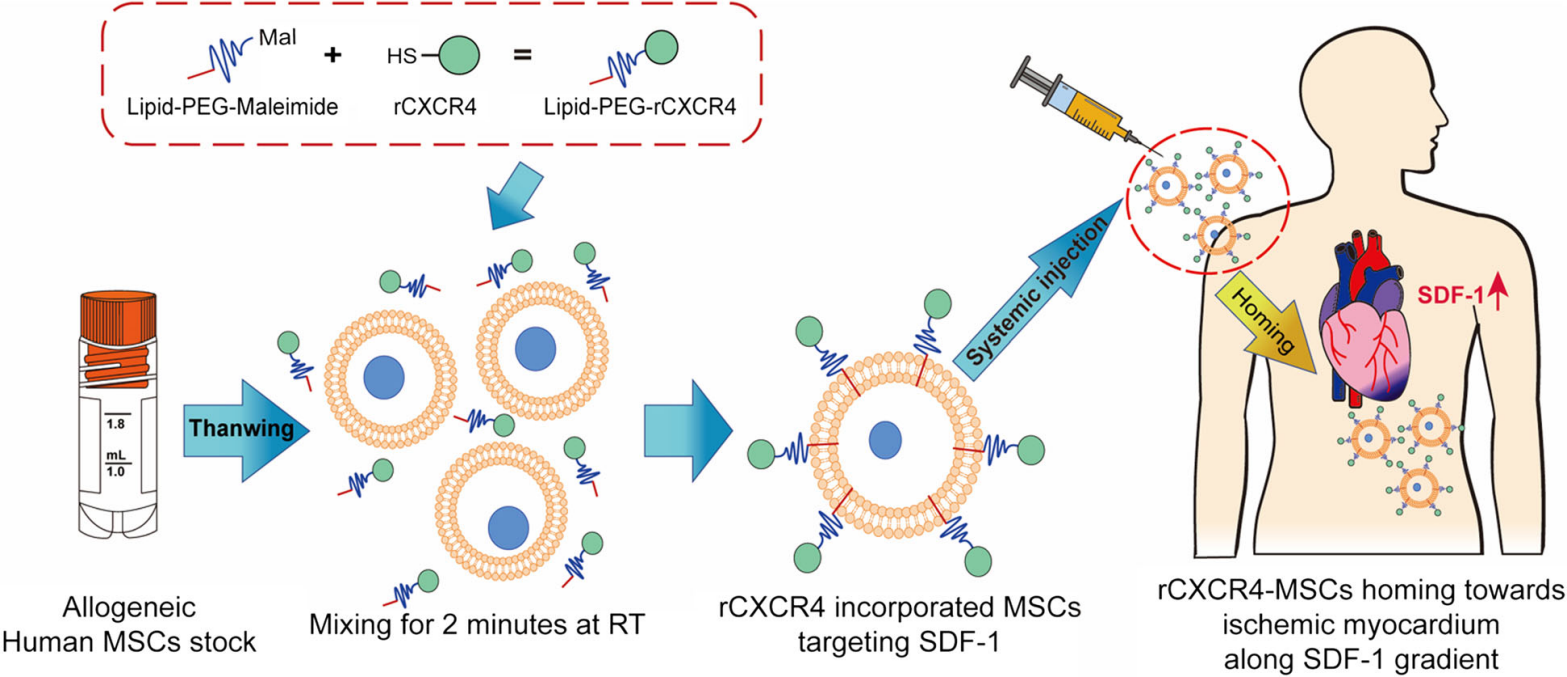
Schematic representation of surface-engineered MSCs for cardiac regeneration. *Ex vivo* cultured and expanded MSCs were surface engineered through hydrophobic insertion to incorporate rCXCR4 on their membrane. Hydrophobic insertion generated homogeneous MSCs modified with hydrophobized rCXCR4 within a short time. Systemically infused rCXCR4-modified MSCs can migrate to the ischemic myocardium by taking advantage of SDF-1 concentration gradient. Abbreviations: MSCs: mesenchymal stem cells; PEG: Poly(ethylene glycol); rCXCR4: Recombinant CXC chemokine receptor 4; SDF-1: Stromal-derived factor-1

Previously, CXCR4 expression on MSCs had been induced by hypoxic culture conditions, addition of cytokine cocktails, and viral gene transduction. However, these methods are now discouraged due to the lengthy generation time and risk of altering the MSC properties [12, 133–135]. In order to exploit the SDF-1 gradient for targeted delivery of MSCs to the MI site, pre-expanded MSCs should be rapidly modified with the targeting moiety. Cell surface engineering using the hydrophobic insertions provides an excellent solution to enhance the homing of MSCs to the injured myocardium. Because it noninvasively engineers cells and readily modifies the cell membrane with therapeutic molecules containing lipophilic anchors [136], cell modification by hydrophobic insertion allows instantaneous generation of specialized therapeutic MSCs without a detrimental effect. To demonstrate the feasibility, MSCs were surface-engineered with recombinant CXCR4 (rCXCR4) hydrophobized with DMPE-PEGs (Fig. 2) [43]. In less than 10 min of incubating pre-expanded MSCs with rCXCR4-PEG-DMPE, specialized MSCs were generated. These cells exhibited a recovered response to SDF-1 with a two-fold improvement of migration ability toward the concentration gradient of SDF-1. Thus, cell surface engineering of MSCs with rCXCR4-PEG-DMPE will be explored with a focus on approaches that further enhance the therapeutic potential of MSCs for regenerative medicine.

## Conclusion

Cell therapy has advanced to the point where it aims to provide treatments for tissue degeneration, chronic inflammation, autoimmunity, genetic disorders, cancer, and infectious diseases [84]. Because the efficacy of cell therapy heavily depends on manipulating the fate and function of therapeutic cells, innovative strategies are continuously being introduced to enhance cell survival, boost native behaviors, add new functions, and improve therapeutic effects. Genetic modification has the advantage of expressing heterologous proteins in cells; however, the expression of desired protein heavily depends on the amount of genetic materials internalized by the cells and the efficiency of protein synthesis of the targeted cells. Nonetheless, viral gene transfer limits the application of genetically modified cells for therapies due to safety and economical concerns, including the use of viral vectors, expensive production cost, and extensive generation time. Non-genetic engineering allows for more creative designs to redirect cells for therapeutic purposes. Both synthetic and natural biomaterials can be incorporated onto the cell surface through covalent conjugation, electrostatic interaction, and hydrophobic interaction in order to provide unique properties and functionalities to cells. Although covalent conjugation and electrostatic interaction provide stable surface modification, the degree of modification is difficult to control. Excessive modification may disrupt the membrane integrity, resulting in severe cytotoxicity. Compared to other surface engineering methods, hydrophobic interaction is a safer membrane modification method that noninvasively modifies the cell surface by inserting lipid-conjugated molecules into the membrane. Despite limited understanding of the fate of lipid conjugated bioactive substances, surface engineering with hydrophobic interaction is an attractive technique because it can be applied to virtually any cell. Non-genetic cell surface engineering to improve their therapeutic potentials is still in its infancy, suggesting each technology should be further tailored to overcome the disadvantage and meet the specific demands of clinical application.

## Abbreviations

AMI: Acute myocardial infarction
BCP-ALL: B cell precursor acute lymphoblastic leukemia
bFGF: Basic fibroblast growth factor
CARs: Chimeric antigen receptors
CD19: Cluster of differentiation 19
CXCR4: CXC chemokine receptor 4
DiD: 1,1’-dioctadecyl-3,3,3’,3’- tetramethylindodicarbocyanine, 4-chlorobenzenesulfonate salt
Dil: (1,1’-dioctadecyl-3,3,3’ ,3’-tetramethylindocarbocyanine perchlorate)
DiO: 3,3’ -dioctadecyloxacarbocyanine, perchlorate
DiR: 1,1’-dioctadecyltetramethyl indotricarbocyanine Iodide
DMPE: 1,2-dimyristoyl-sn-glycerol-3-phosphatidylethanolamine
DPPE: 1,2-dipalmitoyl-sn-glycerol-3-phosphatidylethanolamine
DSPE: 1,2-distearoyl-sn-glycerol-3-phosphatidylethanolamine
FDA: Food and Drug Administration
FGF2: Fibroblast growth factor-2
FITC: Fluorescein isothiocyanate
FRAP: Fluorescent recovery after photobleaching
GVHD: Graft-versus-host disease
HA: Hyaluronic acid
HO-1: Heme oxygenase-1
HSCs: Hematopoietic stem cells
IL: Interleukin
iPSCs: Induced pluripotent stem cells
ManNAz: *N*-α-azidoacetylmannosamine
MCP-1: Monocyte chemoattractant protein-1
MHC: Major histocompatibility complex
MI: Myocardial infarction
MSCs: Mesenchymal stem cells
NHS: *N*-hydroxyl-succinimidyl ester
NK cells: Natural Killer cells
PAA: Poly(acrylic acid)
PAH: Poly(allylamine hydrochloride)
PDADMAC or PDDA: Poly(diallydimethylammonium) chloride
PEG: Poly(ethylene glycol)
PEI: Poly(ethyleneimine)
PIGF: Placental growth factor (PIGF)
PLL: Poly-L-lysine
PLL-g-PEG: PLL-graft-poly(ethylene glycol)
PPP: Polyphosphoric acid
PSS: Poly(styrene) sulfate
PVA: Poly(vinyl alcohol)
rCXCR4: Recombinant CXC chemokine receptor 4
SDF-1: Stromal-derived factor-1
SiaNAz: *N*-α-azidoacetyl sialic acid
SPR: Surface plasmon resonance
TILs: Tumor infiltrating lymphocytes
VEGF: Vascular endothelial growth factor
